# Effectiveness of First Aid Training at Home for Grandparents Caring Grandchildren Under 5 Years Old: A Randomized Controlled Trial

**DOI:** 10.1155/tswj/7457823

**Published:** 2025-09-02

**Authors:** Atefeh Rahim, Abdolrahim Asadollahi, Mehdi Mojadam, Eva Dolenc Šparovec, Mansour Kashfi, Mahin Nazari

**Affiliations:** ^1^Student Research Committee, Shiraz University of Medical Sciences, Shiraz, Iran; ^2^Department of Gerontology, School of Health, Shiraz University of Medical Sciences, Shiraz, Iran; ^3^School of Health, Ahvaz Jondishapur University of Medical Sciences, Ahvaz, Iran; ^4^Public Health Division, Faculty of Health Sciences, University of Ljubljana, Ljubljana, Slovenia; ^5^Department of Public Health, School of Health, Shiraz University of Medical Sciences, Shiraz, Iran; ^6^Department of Health Promotion, School of Health, Shiraz University of Medical Sciences, Shiraz, Iran

**Keywords:** educational intervention, first aid, grandchild care, home accidents, knowledge, older parents

## Abstract

**Background:** This study is aimed at raising awareness about home accidents among grandparents caring for their grandchildren and reducing the risk of accidents and premature death in children under five through structured first aid training.

**Methods:** In this randomized controlled trial, 76 older adults from a daycare center in Shiraz, Iran, were randomly assigned to either an intervention group or a control group. The intervention group participated in eight sessions of first aid training. Pre- and postintervention assessments included PMT-based questionnaires and practical first aid skill tests. Data were analyzed using SPSS and JAMOVI.

**Results:** Significant improvements were observed in the intervention group compared to the control group in terms of first aid knowledge (*p* < 0.001), home accident prevention (*p* < 0.001), and practical first aid skills (*p* < 0.001). Effect size analyses (Cohen's *d* > 0.80) further supported the substantial impact of the intervention across all measured domains.

**Conclusions:** First aid training based on protection motivation theory significantly enhanced older adults' preparedness in preventing and managing home accidents involving their grandchildren. These findings underscore the importance of integrating first aid education into geriatric caregiving programs.

**Trial Registration:** Iranian Registry of Clinical Trials number: IRCT20180514039648N6.

## 1. Introduction

Globally, home accidents are a leading cause of preventable disabilities and death among children and adolescents [1]. They are among major public health problems, which can be prevented by taking simple preventive measures both at home and in the neighborhood. For this purpose, families should be informed about and trained on how to prevent home accidents [2]. The World Health Organization (WHO) defines an accident as an event “that occurs unwillingly and causes physical and mental damage by sudden external force” [1]. Currently, accidents rank highest among preventable health problems across all age groups, particularly in childhood in developed and developing countries, and the leading causes of death and disability [2]. In modern times, children have to deal with the risks brought on by technology. In addition to creating a multitude of electrical household appliances, congested streets, and skyscrapers, technological advancements have also increased the number of causes of death and disability [3]. Surveys have shown that children and adolescents are most susceptible to risks and accidents [4]. Due to their disproportionate amount of time spent indoors, children under the age of five are more vulnerable to injuries sustained at home (household injuries) [5]. Although home is a safe place for children, nearly one-third of accidents occur there [6]. Children under the age of five frequently survive accidents that cause physical or mental damage that restricts their activities in the long run [1]. Accidental trauma is the second leading cause of disability in developing countries and the third leading cause of death and disability worldwide. Burns, poisoning, falls, and trauma are the four leading causes of death among children under the age of five globally [4]. Infants and young children are susceptible to serious injuries and accidents, which can be largely prevented by acquiring the right knowledge and safe practices [7]. Children need adult supervision to provide them with a safe environment and shield them from accidents [2]. With many mothers working outside the home, the older persons can increasingly contribute to the health and well-being of the third generation.|They can also serve as a valuable source of knowledge and experience for their grandchildren [8] and are increasingly involved in their care and protection [9]. Although they have always been vital members of the family, many of them have assumed greater responsibility for their grandchildren over the past two decades as a result of social and familial changes and problems. The first major change is the provision of childcare [10]. In contemporary society, grandparents take a proactive role and provide hands-on care for their grandchildren, freeing up parents to engage in personal and professional activities [11]. It can be fulfilling for both generations to have contact with their grandparents. Unlike working parents, grandparents typically do not have a regular job and have ample time to care for their grandchildren [10]. Most home accidents can be prevented by raising awareness, improving the home environment, and increasing safety [7]. The difficulty lies in ensuring that everyone is trained to administer life-saving first aid in a medical emergency [12]. Many accidents can be prevented if child caregivers adopt proper safety measures [7].

## 2. Methodology

### 2.1. Study Design and Population

The present study was a randomized controlled trial with an international registration code conducted in 2023–2024. The sample size was determined using NCSS PASS Version 15. The impact factor of the research model was calculated to be 0.939 at a confidence level of 95% and a significance level of 0.05. Given a squared standard deviation of 0.33 in similar studies (e.g., Eva Dolenc, 2021), the sample size, including a 15% drop in the number of subjects, was determined to be 76. The participants were divided into two groups of 38 using a random number table in Excel [13]. This study was conducted in the Farzanegan Daily Caring Foundation (FDCF) in Shiraz, which serves a large number of older people and provides daily education and care for them, making it an ideal location for trials and planned interventions. (It is a nongovernmental organization that offers daycare services to the older adults and is officially licensed in provincial centers by the State Welfare Organization of Iran). The inclusion criteria included willingness and informed consent to participate in the study, literacy, caring for grandchildren under 5 years of age, and caring for grandchildren in the absence of parents. The exclusion criteria, on the other hand, included not responding to survey questions, contracting a disease affecting childcare, unwillingness to further cooperate, not responding to posttest questions, and not caring for grandchildren during the study period.

### 2.2. Data Collection

The researcher gave the grandparents a detailed explanation of the research objectives and methodology after receiving their written informed consent. The participants were then asked to complete four questionnaires and administer a skill assessment practice test. The research questionnaires included: (1) a demographic questionnaire to measure the standard demographic indicators of the study population with 12 questions (items) covering variables such as age, gender, level of education, marital status, grandchild's gender, (taking) responsibility for grandchildren, (taking) responsibility of taking grandchildren to school, suffering from a chronic illness, illness type and duration, grandchild's illness and disability, and health status. (2) Older Adults' First Aid Knowledge Scale (OFAKS, 18-item) including 12 items, six on the first aid knowledge and six on the attitudes towards first aid. It was designed based on previous empirical research on various aspects of first aid, which included four sets of basic questions: first aid awareness, willingness to provide first aid, attending previous first aid training sessions, and willingness to participate in additional courses [13]. It was conducted using the WHO back-translation method to validate the aging population. (3) A 31-item accident prevention questionnaire based on the constructs of the protection motivation theory (PMT). The questions were rated on a 5-point Likert scale: I strongly disagree (1 point), I disagree (2 points), I neither agree nor disagree (3 points), I agree (4 points), and I strongly agree (5 points). The PMT construct was also assessed using the following question: “Have you ever thought of adopting measures to prevent home accidents involving your child?”, which measures grandparents' intentions of adopting [proper] measures to prevent home accidents. (4) Skills Assessment Checklist to assess pre- and postintervention practical first aid (management) skills of older parents, which included their skills in burns, poisoning, falls, first aid, and basic resuscitation of children. The checklist includes five questions ranging from 0 (*no skills*) to 10 (*full skills*). (5) A first aid practice test conducted by a researcher and an emergency specialist to measure the skills acquired by the older parents.

### 2.3. Procedure and Intervention

Individuals who met the inclusion criteria were invited to attend the training sessions. The pretest questionnaires were distributed among the intervention and control groups during a separate briefing session. The intervention group was then invited to attend the next sessions and received the required training. For those in the intervention group, the educational intervention comprised eight 60-min sessions, including lectures and question and answer (Q&A) sessions, photo and video demonstrations, educational pamphlet distribution, hands-on application by an emergency specialist, and PowerPoint presentations. Finally, at the conclusion of the 2-month training, the posttest questionnaires were distributed among participants. The control group also received the questionnaires and completed them at the same time as the intervention group. Educational pamphlets were then distributed to the control group members after the sessions to ensure compliance with ethical standards.

### 2.4. Statistical Analysis

The Shapiro–Wilk and Kolmogorov–Smirnov tests were used to measure the data, which displayed a normal distribution. The questionnaires' validity was evaluated using the McDonald's omega test and Cronbach's alpha, which was acceptable. Independent and paired-samples *t*-tests were performed to determine the difference between the groups before and after the intervention. The demographic data was statistically analyzed using the chi-squared test, mean, and standard deviation. The intervention effect sizes were Cohen's *d*, Glass' delta, and Hedges' *G*. The research effectiveness was also determined using Cohen's U3. Data analysis was performed using SPSS v.28 at a significance level of 0.05 and JAMOVI v.2.3 (2023) [14, 15].

### 2.5. Ethical Considerations

**Figure 1:**
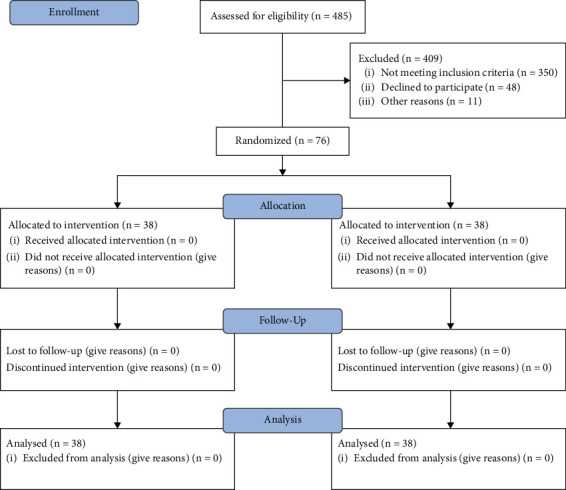
Flow diagram for study participants.

The study protocol was approved by the Ethics Committee of Shiraz University of Medical Sciences (IR.SUMS.SCHEANUT.REC.1402.009), and all procedures were conducted in accordance with the Declaration of Helsinki (2013 revision) and the CONSORT 2010 statement for randomized controlled trials (see Figure 1). All participants were informed of the study's objectives and provided written informed consent. Confidentiality and anonymity were assured throughout the research process.

## 3. Findings

### 3.1. Demographic Data

In this study, a total of 76 older parents were examined, with a mean age of 66.84 ± 2.756 years in the intervention group and 67.32 ± 2.742 years in the control group, according to a review of their general profile.  Table 1 indicates no significant difference between the experimental and control groups in terms of demographic variables, such as chronic diseases, disease duration, ethnicity, gender, marital status, level of education, grandchild's gender, (taking) responsibility for grandchildren, health status, and so forth (*p* > 0.05). According to the distribution of diseases, “none” was the most often selected option in the intervention group, while “comorbidity” was the most frequently selected option in the control group.

### 3.2. Statistical Analysis

According to Table 2, no significant difference could be found between the intervention and control groups for the accident prevention scores before the educational intervention. Following the intervention, however, the mean accident prevention scores were significantly higher in the intervention group than in the control group. A within-group comparison revealed a significant increase in the mean accident prevention scores of the intervention group after implementing the educational intervention on the aging population under study. However, the control group showed a significant difference in this index before and after the intervention (*p* ≤ 0.001). This difference can be attributed to the Hawthorne effect, that is, when subjects of an experimental study attempt to change or improve their behavior simply because it is being evaluated or studied.

Table 2 lists the mean accident prevention scores before and after the intervention in the intervention and control groups. A between-group comparison revealed no significant difference between the two groups in the mean accident prevention scores before the educational intervention (*p* > 0.05). Following the intervention, however, the mean accident prevention scores were significantly higher in the intervention group than in the control group (*p* ≤ 0.001). A within-group comparison revealed a significant increase in the mean accident prevention scores of the intervention group after implementing the educational intervention on the aging population under study. However, the control group showed a significant difference in this index before and after the intervention (*p* ≤ 0.001). This difference can be attributed to the Hawthorne effect, that is, when subjects of an experimental study attempt to change or improve their behavior simply because it is being evaluated or studied. The research effect sizes were obtained at a significance level of < 0.001, > 1, and favorable (Table 2). This indicates a 170% increase in the accident prevention score of the older parents in the intervention group as a result of the educational intervention (Cohen's *d* = 1.70), while it was < 0.05% for those in the control group. Other effect sizes such as Glass' delta and Hedges' *G* also showed favorable results for the intervention group in line with Cohen's *d* (Glass' delta = 1.9 and Hedges' *G* = 1.77). Cohen's U3 also indicated that the physical activity performed by the studied aging population could increase their accident prevention score by 85.8%. This effect size was in the [0, 1] range expressed in percentage, where 1 means 100% intervention effectiveness.

**Figure 2:**
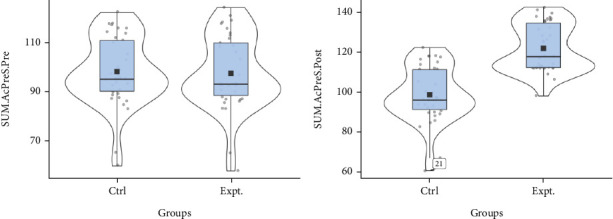
Violin diagram of the distribution of the total mean of the AcPreS variable before and after the intervention in two groups.

Figure 2 depicts a violin plot of the total home accident prevention score to determine the difference over time between the intervention and control aging parent groups in two time series (clockwise rotation). The average line in the intervention group indicated a significant increase in the two measurement time points compared to the control group, confirming an increase in the home accident prevention score of the studied older parents' grandchildren. A violin plot is a fusion of a box plot and a kernel density plot, which displays peaks and distributions in the data. For the samples with multiple mode peaks, the violin plot clearly depicts data density, multiple peak points, and their coordinates and relative fluctuations, especially in studies with multiple measurement time points. These are the most important advantages of violin plots over box plots and histograms [14].

Table 3 lists the mean first-aid awareness scores of the intervention and control groups before and after the intervention. No significant difference could be found between the two groups for the mean first-aid awareness scores before the educational intervention (*p* > 0.05). Following the intervention, however, the mean first-aid awareness score of the intervention group was significantly higher than that of the control group (*p* ≤ 0.001). A within-group comparison revealed that following the educational intervention in the aging population under study, the intervention group's mean first aid awareness scores significantly increased while the control group's scores did not change significantly. The research effect sizes were obtained at a significance level of < 0.001, > 1, and favorable (Table 3). This indicates a 173% increase in the first aid awareness score of the older parents in the intervention group as a result of the educational intervention (Cohen's *d* = 1.73), while it was < 0.05% for those in the control group. Other effect sizes such as Glass' delta and Hedges' *G* also showed favorable results for the intervention group in line with Cohen's *d* (Glass' delta = 1.6 and Hedges' *G* = 1.52). Cohen's U3 also indicated that the educational intervention in the studied aging population could increase their first aid awareness score by 85.8%.

**Figure 3:**
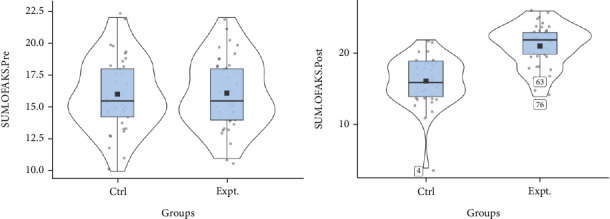
Violin diagram of the distribution of the total mean of OFAKS variable before and after the intervention in two groups.

Figure 3 depicts a violin plot of the total first-aid awareness score to determine the difference over time between the intervention and control older parent groups in two time series (clockwise rotation). The average line in the intervention group indicated a significant increase in the two measurement time points compared to the control group, confirming an increase in the first aid awareness score of the studied older parents.

Table 4 lists the mean scores of the first-aid practice test on child's moulage by the older parents before and after the intervention in the intervention and control groups. A between-group comparison revealed no significant difference between the two groups for the older parents' mean first-aid practice test scores before the educational intervention (*p* > 0.05). Following the intervention, however, the mean first aid practice test score of the intervention group was significantly higher than that of the control group (*p* ≤ 0.001). A within-group comparison revealed that following the educational intervention in the aging population under study, the intervention group's mean first aid practice test scores significantly increased while the control group's scores did not change significantly (*p* ≤ 0.001). The research effect sizes were obtained at a significance level of < 0.001, > 1, and favorable (Table 5). This indicates a 101% increase in the first aid skills score of the older parents in the intervention group as a result of the (first aid) educational intervention (Cohen's *d* = 1.01), while it was < 0.05% for those in the control group. Other effect sizes such as Glass' delta and Hedges' *G* also showed favorable results for the intervention group in line with Cohen's *d* (Glass' delta = 2.4 and Hedges' *G* = 1.80). Cohen's U3 also indicated that the first aid training courses in the studied aging population could increase their first-aid skills score by 84.4%.

**Figure 4:**
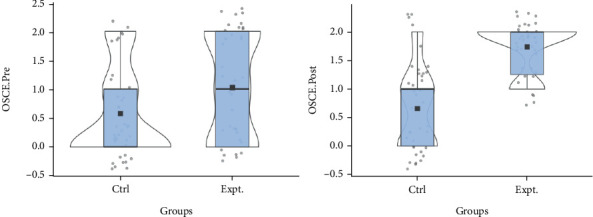
Violin diagram of the distribution of the total mean of OSCE variable before and after the intervention in two groups.

Figure 4 depicts a violin plot of the total score of the first aid practice test on a child's moulage by the studied older parents to determine the difference over time between the intervention and the control older parent groups in two time series (clockwise rotation). The average line in the intervention group indicated a significant increase in the two measurement time points compared to the control group, confirming an increase in the practical first-aid skills score of the studied older parents.

Table 5 lists the mean first aid skills scores of the older parents before and after the intervention in the intervention and control groups. A between-group comparison revealed no significant difference can be found between the two groups for the mean first aid skills scores before the educational intervention (*p* > 0.05). Following the intervention, however, the mean first aid skills score of the intervention group was significantly higher than that of the control group (*p* ≤ 0.001). A within-group comparison revealed that following the educational intervention in the aging population under study, the intervention group's mean first aid skills scores significantly increased (*p* ≤ 0.001), while the control group's scores did not change significantly. The research effect sizes were obtained at a significance level of < 0.001, > 1, and favorable (Table 5). This indicates an 82% increase in the first aid skills score of the older parents in the intervention group as a result of the (first aid skills) educational intervention (Cohen's *d* = 0.81), while it was < 0.05% for those in the control group. Other effect sizes such as Glass' delta and Hedges' *G* also showed favorable results for the intervention group in line with Cohen's *d* (Glass' delta = 0.85 and Hedges' *G* = 0.95). Cohen's U3 also indicated that the first-aid training courses in the studied aging population could increase their first aid skills score by 79.4%.

**Figure 5:**
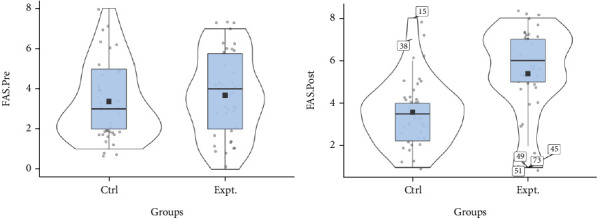
Violin diagram of the distribution of FAS variable before and after the intervention in two groups.

Figure 5 depicts a violin plot of the total practical first aid skills score of the studied older parents to determine the difference over time between the intervention and the control older parent groups in two time series (clockwise rotation). The average line in the intervention group indicated a significant increase in the two measurement time points compared to the control group, confirming an increase in the practical first aid skills score of the studied older parents.

## 4. Discussion

This randomized controlled trial demonstrated that structured first aid training significantly improves grandparents' (older parents') awareness, skills, and attitudes regarding home accident prevention. The findings align with existing international evidence while also contributing novel insights from the Iranian sociocultural context.

### 4.1. Improvement in First Aid Knowledge and Awareness

The significant increase in first aid knowledge following the intervention (Cohen's *d* = 1.73) confirms the potential of tailored educational programs for older caregivers. These results are consistent with the findings of Dolenc et al., who emphasized that older adults have both the motivation and capacity to learn first aid when content is adapted to their cognitive and physical capabilities [13]. Similarly, Ammirati et al. stressed that even young children can be trained in first aid if the training is age-appropriate, which indirectly supports the premise that educational content should be adapted to learner characteristics—an approach this study followed [12].

### 4.2. Enhancement of Practical First Aid Skills

The postintervention improvement in practical skills (Cohen's *d* = 1.01) suggests that older adults not only retain cognitive knowledge but also translate it into action. This challenges common assumptions that aging significantly limits learning capacity in procedural domains. Our results corroborate the findings of Dolenc et al., who demonstrated that hands-on training, especially involving moulage and scenario-based learning, was effective in older adult populations [16, 17]. In the Iranian context, where caregiving is increasingly delegated to grandparents, these findings are highly relevant.

### 4.3. Increased Readiness for Home Accident Prevention

A notable increase in home accident prevention behaviors and intentions (Cohen's *d* = 1.70) was also observed. The intervention utilized PMT, which proved effective in altering risk perception and self-efficacy. Prior Iranian studies (e.g., [18]) also support the use of PMT-based educational interventions to improve safety behaviors among parents of young children [6]. However, our study is the first to apply PMT to a senior population caring for grandchildren, which adds to the theoretical scope of PMT applications.

### 4.4. Cultural and Policy Implications

This study addresses a growing caregiving trend in Iran and similar societies where grandparents serve as primary caregivers. While previous literature [9] has explored the caregiving role of grandparents in Western settings, little has been done in Middle Eastern or Iranian contexts. The results point to the need for national health policy reforms that recognize older adults as active contributors to child health, especially in intergenerational households.

### 4.5. Limitations and Suggestions for Future Research

This study had the following limitations: A larger sample size for this study would have been preferable to increase productivity and yield more desirable results. The educational workshops were kept general due to time constraints and the older parents' impatience. More comprehensive workshops with separate sessions on burns, choking, falls, and so forth would have been preferable. The intervention was conducted in the fall when the flu pandemic made it challenging to encourage the older persons to attend the training sessions. It was also challenging to find and reach a sample that met certain inclusion criteria (e.g., caring for a grandchild under the age of five).

## 5. Conclusion

The findings demonstrated that providing older parents with first aid training significantly enhances their ability to prevent and manage home accidents involving their grandchildren. This highlights the importance of integrating targeted educational programs into caregiving practices to improve child safety. Such initiatives can effectively reduce the risk of home accidents and contribute to better health outcomes for grandchildren under their car.

## Figures and Tables

**Table 1:** Characteristics of demographic variables between groups.

**Variables**	**Classification**	**Groups**				**Sig.**
		**Experiment **(**n** = 38)		**Control (** **n** = 38**)**		
		**Frequency**	**Percent**	**Frequency**	**Percent**	
Sex	Female	31	81.6	26	68.4	0.251
	Male	7	18.4	12	31.6	

Nationality	Persians	32	84.2	27	71.1	0.425
	Lors	2	5.3	5	13.2	
	Turks	1	2.6	5	13.2	
	Balochis	3	7.9	1	2.6	

Marital status	Married	23	60.5	23	60.5	0.452
	Widow	11	28.9	15	39.5	
	Divorced	4	10.5	0	0	

Level of education	Graduated	31	81.6	34	89.5	0.381
	Diploma and less	7	18.4	4	10.5	

Gender of grandchildren	Girl	23	60.5	14	36.8	0.228
	Boy	12	31.6	20	52.6	
	Both	3	7.9	4	10.5	

Responsibility for grandchildren	Nutrition	1	2.6	0	0	0.365
	Bathing	1	2.6	0	0	
	Changing clothes	0	0	0	0	
	Just care	23	60.5	18	47.4	
	Circulation	2	5.3	2	5.3	
	Deliver to school	2	2.6	1	2.6	
	All items	10	26.3	17	44.7	

The responsibility of sending grandchildren to school and kindergarten	Father	5	13.2	4	10.5	0.302
	Mother	4	10.5	6	15.8	
	Both in turn	12	31.6	11	28.9	
	By my own	6	15.8	3	7.9	
	Other	11	28.9	14	36.8	

Having a chronic disease	Yes	21	55.3	34	89.5	0.058
	No	17	44.7	4	10.5	

Duration of chronic illness	Does not have	17	44.7	4	10.5	0.324
	Less than a year	1	2.6	1	13.2	
	2–5 years	4	10.5	14	10.5	
	6–10 years	9	23.7	12	5.3	
	<11 years	7	18.4	7	5.3	

Illness & disability in grandchildren	Yes	6	15.8	3	0	0.189
	No	32	84.2	35	44.7	

Health condition	Yes	20	52.6	9	10.5	0.239
	No	18	47.4	29	10.5	

*Note:* Significance less than 0.05.

**Table 2:** Comparison of mean score of AcPreS before and after the intervention between two groups.

**AcPreS study variable**	**Before (** **n** = 38**)**		**After (** **n** = 38**)**		**Student's ** **t** **-test** ^**2**^	**ES**	
	**Mean**	**SD**	**Mean**	**SD**		**Cohen's ** **d**	**Cohen's U3** ^**c**^
Control	98.11	13.928	98.71	13.715	*t* (76) = 0.001	—	—
Experiment	97.42	14.857	121.61	11.804	*t* (76) = 0.001^∗∗∗^	1.703	85.8
Student's *t*-test^1^	*t* (38) = 0/836^n.s.^		*t* (38) = 0001^∗^				
ES				
Cohen's *d*	0.048		1.789	
Glass' delta^a^	0.046		1.940	
Hedges' *G*^b^	0.047		1.771	

Abbreviations: ES, effect size; n.s., not statistically significant.

^1^Statistical independent *t*-test.

^2^Statistical paired *t*-test, DF ≥ 69.

^a^Each group has a different standard deviation.

^b^Each group has a different sample sizes.

^c^% of the “treatment” group will be above the mean of the “control” group.

⁣^∗∗∗^Statistically significant at the 1% level (*p* value ≤ 0.001).

**Table 3:** Comparison of the average index scores of OFAKS before and after the intervention.

**OFAKS study variable**	**Before (** **n** = 38**)**		**After (** **n** = 38**)**		**Student's ** **t** **-test** ^**2**^	**ES**	
	**Mean**	**Effect coefficients**	**Mean**	**Effect coefficients**		**Cohen's ** **d**	**Cohen's U3** ^**c**^
Control	16.03	2.852	16.21	3.370	*t* (76) = 0.563^n.s.^	—	—
Experiment	16.11	2.788	21.13	2.997	*t* (76) = 0.001^∗∗∗^	1.735	85.8
Student's *t*-test^1^	*t* (38) = 0.903^n.s.^		*t* (38) = 0.001^∗^				
ES				
Cohen's *d*	0.028		1.543	
Glass' delta^a^	0.028		1.642	
Hedges' *G*^b^	0.028		1.527	

Abbreviations: ES, effect size; n.s., not statistically significant.

^a^Each group has a different standard deviation.

^b^Each group has a different sample size.

^c^% of the “treatment” group will be above the mean of the “control” group.

^1^Statistical independent *t*-test.

^2^Statistical paired *t*-test, DF ≥ 69.

⁣^∗∗∗^Statistically significant at the 1% level (*p* value ≤ 0.001).

**Table 4:** Comparison of the average index of the OSCE before and after the intervention.

**OSCE study variable**	**Before (** **n** = 38**)**		**After (** **n** = 38**)**		**Student's ** **t** **-test** ^**2**^	**ES**	
	**Mean**	**Standard deviation**	**Mean**	**Standard deviation**		**Cohen's ** **d**	**Cohen's U3** ^**c**^
Control	0.58	0.793	0.66	0.708	*t* (76) = 0.474^n.s.^	—	—
Experiment	1.03	0.885	1.74	0.446	*t* (76) = 0.001^∗∗∗^	1.012	84.4
Student's *t*-test^1^	*t* (38) = 0.053^n.s.^		*t* (38) = 0.001^∗^				
ES				
Cohen's *d*	0.532		1.823	
Glass' delta^a^	0.506		2.418	
Hedges' *G*^b^	0.527		1.804	

Abbreviations: ES, effect size; n.s., not statistically significant.

^a^Each group has a different standard deviation.

^b^Each group has a different sample size.

^c^% of the “treatment” group will be above the mean of the “control” group.

^1^Statistical independent *t*-test.

^2^Statistical paired *t*-test; DF ≥ 69.

⁣^∗∗∗^Statistically significant at the 1% level (*p* value ≤ 0.001).

**Table 5:** Comparison of the average score index in FAS before and after the intervention.

**FAS study variable**	**Before (** **n** = 38**)**		**After (** **n** = 38**)**		**Student's ** **t** **-test** ^**2**^	**ES**	
	**Mean**	**Standard deviation**	**Mean**	**Standard deviation**		**Cohen's ** **d**	**Cohen's U3** ^**c**^
Control	3.37	1.909	3.58	1.605	*t* (76) = 0.457^n.s.^	—	—
Experiment	3.68	2.055	5.39	2.125	*t* (76) = 0.001^∗∗∗^	0.818	79.4
Student's *t*-test^1^	*t* (38) = 0.490^n.s.^		*t* (38) = 0.001^∗^				
ES				
Cohen's *d*	0.195		0.964	
Glass' delta^a^	0.154		0.854	
Hedges' *G*^b^	0.158		0.995	

Abbreviations: ES, effect size; n.s., not statistically significant.

^.a^Each group has a different standard deviation.

^b^Each group has a different sample size.

^c^% of the “treatment” group will be above the mean of the “control” group.

^1^Statistical independent *t*-test.

^2^Statistical paired *t*-test; DF ≥ 69.

⁣^∗∗∗^Statistically significant at the 1% level (*p* value ≤ 0.001).

## Data Availability

The datasets generated and/or analyzed during the current study are available from the authors upon reasonable request and with the permission of SUMS.
